# Dose of aspirin to prevent preterm preeclampsia in women with moderate or high-risk factors: A systematic review and meta-analysis

**DOI:** 10.1371/journal.pone.0247782

**Published:** 2021-03-09

**Authors:** Rachel Van Doorn, Narmin Mukhtarova, Ian P. Flyke, Michael Lasarev, KyungMann Kim, Charles H. Hennekens, Kara K. Hoppe

**Affiliations:** 1 University of Wisconsin-Madison School of Medicine and Public Health, Madison, Wisconsin, United States of America; 2 Department of Obstetrics and Gynecology, Division of Maternal-Fetal Medicine, University of Wisconsin School of Medicine and Public Health, Madison, Wisconsin, United States of America; 3 Department of Biostatistics and Medical Informatics, University of Wisconsin, Madison, Wisconsin, United States of America; 4 Charles E. Schmidt College of Medicine, Florida Atlantic University, Boca Raton, Florida, United States of America; Kobe University Graduate School of Medicine School of Medicine, JAPAN

## Abstract

**Objective:**

To evaluate the effect of aspirin dose on the incidence of all gestational age preeclampsia and preterm preeclampsia.

**Data sources:**

Electronic databases (Cochrane, PubMed, Scopus, ClinicalTrials.gov and the Web of Science) were searched for articles published between January 1985 and March 2019 with no language restrictions.

**Methods:**

We followed the PRIMSA guidelines and utilized Covidence software. Articles were screened by 2 independent reviewers, with discrepancies settled by an independent 3^rd^ party. Study selection criteria were randomized trials comparing aspirin for prevention of all gestational age and preterm preeclampsia to placebo or no antiplatelet treatment in women aged 15–55 years with moderate or high-risk factors according to the list of risk factors from American College of Obstetricians and Gynecologists and United States Preventive Services Task Force guidelines. The quality of trials was assessed using the Cochrane risk of bias tool. The data were pooled using a random-effects meta-analysis comparing aspirin at doses of <81, 81, 100, and 150 mg. Pre-specified outcomes were all gestational age and preterm preeclampsia.

**Results:**

Of 1,609 articles screened, 23 randomized trials, which included 32,370 women, fulfilled the inclusion criteria. In preterm preeclampsia, women assigned at random to 150 mg experienced a significant 62% reduction in risk of preterm preeclampsia (RR = 0.38; 95% CI: 0.20–0.72; P = 0.011). Aspirin doses <150 mg produced no significant reductions. The number needed to treat with 150 mg of aspirin was 39 (95% CI: 23–100). There was a maximum 30% reduction in risk of all gestational age preeclampsia at all aspirin doses.

**Conclusions:**

In this meta-analysis, based on indirect comparisons, aspirin at a dose greater than the current, recommended 81 mg was associated with the highest reduction in preterm preeclampsia. Our meta-analysis is limited due to the deficiency of homogeneous high evidence data available in the literature to date; however, it may be prudent for clinicians to consider that the optimal aspirin dose may be higher than the current guidelines advise. Future research to compare the efficacy aspirin doses greater than 81 mg is recommended.

**Study registration:**

PROSPERO, CRD42019127951 (University of York, UK; http://www.crd.york.ac.uk/PROSPERO/).

## Introduction

Hypertensive disorders of pregnancy are a leading cause of maternal morbidity and mortality worldwide, accounting for more than 70,000 maternal deaths each year [1]. Of all maternal deaths, 10–15% are directly associated with preeclampsia and eclampsia [2]. Aspirin is an inexpensive and widely available drug that has the potential to safely help pregnant women and neonates. While randomized trials have investigated the prophylactic use of aspirin in preventing preeclampsia, the optimal dose remains unclear. Current guidelines endorsed by American College of Obstetricians and Gynecologists (ACOG) and United States Preventive Services Task Force (USPSTF) recommend low-dose aspirin prophylaxis in women at high risk of preeclampsia as well as for women with more than one of several moderate risk factors for preeclampsia [3, 4]. More recent trials have investigated the use of aspirin at a higher dose of 150 mg [5]. In one trial, 150 mg of daily aspirin in pregnant women deemed high-risk for developing preeclampsia, significantly decreased the incidence of preterm preeclampsia when compared to placebo [5]. In a recent systematic review, the authors concluded that aspirin beginning at or before 16 weeks gestation reduces the incidence of preeclampsia and its adverse outcomes among pregnant women and neonates [6]. We, therefore, performed a systematic review and meta-analysis of 23 randomized controlled trials to explore whether aspirin is effective and, if so, the optimal dose for women with moderate or high-risk factors of preeclampsia. Our primary objective was to determine the effect of aspirin dose on the incidence of all gestational age and preterm preeclampsia.

## Methods

### Data sources

This systematic review followed the Preferred Reporting Items for Systematic Reviews and Meta-analysis (PRIMSA) guidelines utilizing the software, Covidence, and registered as CRD42019127951 in the PROSPERO database (University of York, UK; http://www.crd.york.ac.uk/PROSPERO/). Relevant trials were identified through a search of Cochrane Central Register of Controlled Trials, PubMed, Scopus, ClinicalTrials.gov and the Web of Science from January 1985 to March 2019. We did not search prior to 1985 because the first randomized controlled trial studying antiplatelet therapy as prevention for preeclampsia was published in April of that year [7]. References of prior systematic reviews were queried for additional studies. No language restrictions were applied. Medical Subject Heading (MeSH) terms and keyword included: (((("Hypertension"[MeSH]) OR "Blood Pressure"[MeSH]) OR ("Eclampsia"[MeSH] OR "Pre-Eclampsia"[MeSH] OR hypertension OR "high blood pressure" OR hbp OR eclampsia OR pre-eclampsia OR preeclampsia OR "blood pressure")) AND ("Aspirin"[MeSH] OR "Acetylsalicylic acid" OR aspirin)) AND (pregnan* OR gestation*). Approval from an institutional review board was not required for this review.

### Study selection

We conducted a systematic review and meta-analysis of randomized controlled trials that studied the use of aspirin for the prevention of preeclampsia in women aged 15–55 years with any moderate or high-risk factors of preeclampsia according to the list of risk factors from USPSTF and ACOG guidelines. Studies that were quasi-randomized, cluster randomized, or not peer-reviewed were excluded. Trials using a different treatment regimen were similarly excluded. The primary outcome was the effect of aspirin dose on the incidence of all gestational age preeclampsia and preterm preeclampsia.

The titles and abstracts of the searched articles were independently screened by two reviewers (R.VD. and I.F.), with discrepancies addressed by the third reviewer (K.H.). The full text of the selected articles was individually assessed by two reviewers (R.VD. and N.M.) and any conflicts were resolved by the third reviewer (K.H.). Once the final 24 articles were selected for extraction, N.M. and R.VD. extracted relevant data using Covidence software. The software provides comparison of the two reviewers’ extractions and a consensus spreadsheet which requires a specific selection of which extracted data between the two reviewers gets included into the final document. Any disagreement was resolved by the third reviewer K.H. Extracted data was principally focused on our primary outcome and if available, demographic data, adverse maternal, and neonatal outcomes were also extracted. A final collation of data was exported to Microsoft Excel and sent for statistical review and analysis. Of the 24 articles selected for data extraction, one article reported results as percentage values only. Upon contacting the author for raw data, we did not receive a response. This study was then excluded making our total 23 articles.

Each of the 23 articles was subjected to a quality and risk of bias assessment within Covidence. The Covidence software has a built-in Cochrane RoB 1.0 risk of bias assessment tool.^44^ Studies were screened for the following quality measures and bias risks: sequence generation, allocation concealment, blinding of participants and personnel for all outcomes, blinding of outcome assessors for all outcomes, incomplete outcome data for all outcomes, selective outcome reporting, and other sources of bias. Each category previously listed was independently rated as “High”, “Low”, or “Unclear” by the two reviewers. Covidence’s reports of any discrepancies were subsequently voted on for a final rating.

Risk ratios (RR) quantifying aspirin’s effect relative to placebo were estimated using DerSimonian and Laird’s random-effects model, as we anticipated heterogeneity (measured by the I^2^ statistic) would be above 50%. Pooled RRs and supporting 95% confidence intervals (CI) were computed for overall effect of aspirin on two outcomes; all gestational age preeclampsia and preterm preeclampsia and for studies grouped by aspirin dose 1) at most 81 mg versus more than 81 mg and 2) less than 81 mg, exactly 81 mg, exactly 100 mg, and exactly 150 mg for each outcome as well. Publication bias was assessed graphically using contour-enhanced funnel plots [8]. We also conducted a risk difference analyses allowing for a simple additive reduction in risk of preeclampsia with aspirin dosing, which allowed for an estimation of the number needed to treat (NNT) to prevent one case of preeclampsia. Analyses were conducted using Stata version 15.1 (StataCorp LLC, College Station, TX) with the contributed ‘metan’ [9], ‘metabias’ [10], and ‘confunnel’ [11] packages.

## Results

**Fig 1:**
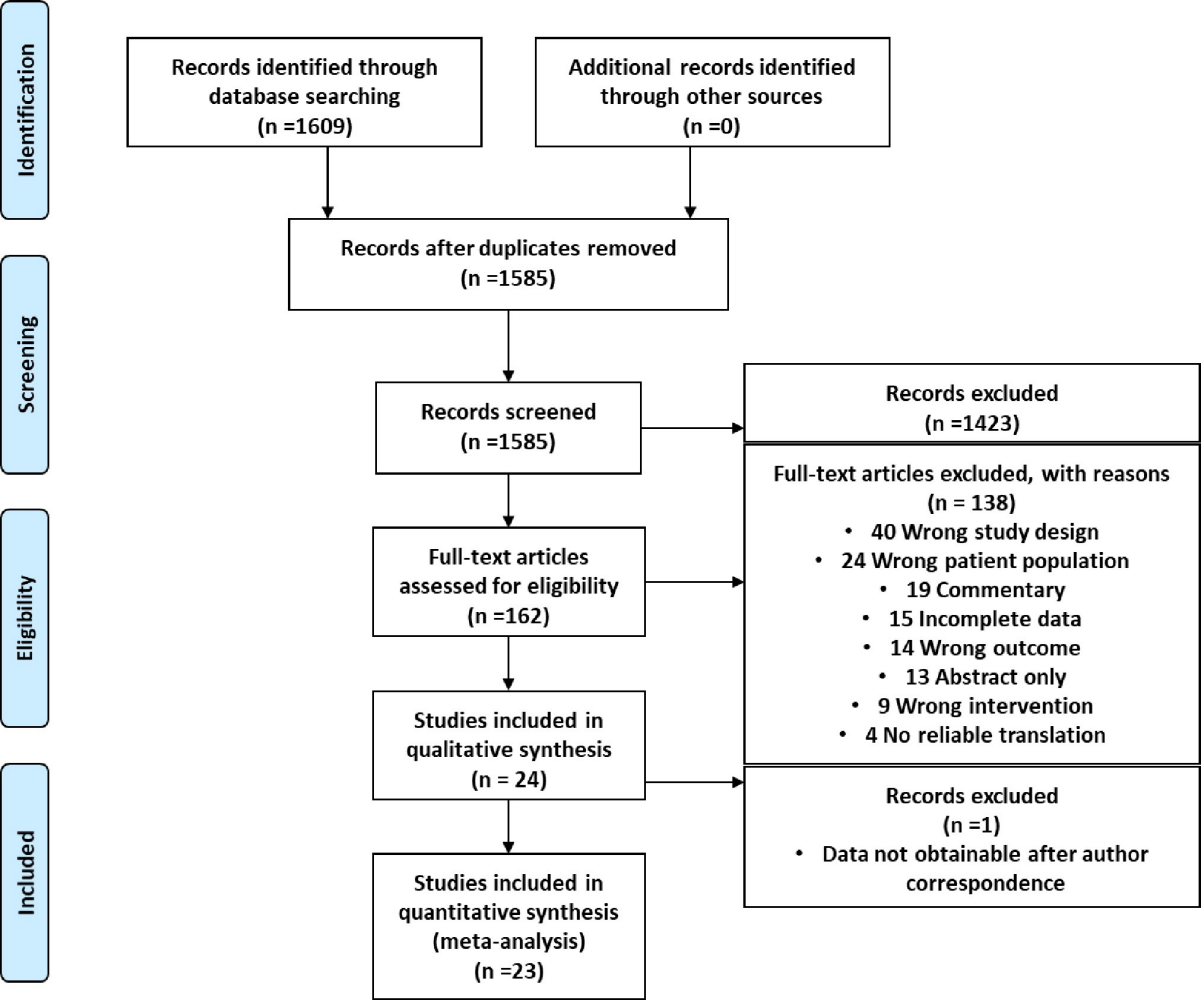
PRISMA flow chart: Summary of article search, selection, and exclusion.

The search strategy yielded 1,609 articles. Preliminary screening by two independent reviewers (R.VD. and I.F.) excluded 24 duplicates. The remaining article titles and abstracts were screened as well as any new articles published during the time of the search. A total of 1,585 potentially eligible manuscripts were identified. Of these manuscripts, 1,423 were excluded for not being relevant to our review criteria. The abstract screening was independently performed by the same reviewers as the initial screen. Lastly, 162 articles went through full-text screening and of those, 138 were excluded for not meeting our inclusion criteria, not having reliable translations, or for being duplicates and commentaries. Any disagreements were settled by the third reviewer (K.H.). Twenty-four articles met the final requirements for inclusion and data was extracted. One of the final 24 studies was excluded for lack of adequate reported data metrics which prevented us from being able to run the proper statistical analyses on their results. One of the remaining 23 articles was only used in preterm preeclampsia analysis. The complete flow diagram of study inclusion is presented in Fig 1.

Study characteristics are presented in Table 1. Our primary outcome measures were the development of preeclampsia as defined by new onset hypertension (systolic blood pressure ≥140 mmHg or diastolic blood pressure ≥90 mmHg on two occasions at least 4 hours apart) in pregnant women >20 weeks of gestation and proteinuria (≥300 mg on 24-hour urine collection, or protein/creatine ≥0.3 mg/dL, or dipstick reading of 2+), or in absence of proteinuria, new onset hypertension accompanied by any of the following: thrombocytopenia, renal insufficiency, pulmonary edema, or impaired liver function [3] and preterm (diagnosed at <37 weeks of gestation) preeclampsia. All studies were randomized controlled trials and ranged from 30 to 9,309 total participants. Preeclampsia event was reported by 22/23 studies, however only 11/23 studies reported the specific incidence of preterm preeclampsia.

**Table 1:** Characteristics of 23 randomized control trials included in the meta-analysis.

Study	N	Inclusion Criteria	Follow-Up Completion	Aspirin	Control	Gestational Age at Entry	Outcome(s)
*Ayala 2013 [17]*	350	Familial or personal history of either GHTN or preeclampsia; CHTN; cardiovascular, endocrine, bleeding, or metabolic disease; personal history of spontaneous abortion; multiple pregnancy; obesity; and adolescent or middle-aged nulliparous pregnancy.	98.8%	100 mg	Placebo	13.5 ± 1.4 weeks	Preeclampsia, preterm delivery, IUGR, and stillbirth.
*Beroyz 1994 [18]*	9,309	For prophylactic entry: history of preeclampsia or IUGR in the previous pregnancy, CHTN, renal disease, or other risk factors as maternal age, family history, or multiple pregnancy.	99.4%	60 mg	Placebo	12–32 weeks	Development of proteinuric preeclampsia, estimated duration of pregnancy, birthweight, birthweight <3^rd^ centile for sex and gestational age, stillbirth, or neonatal death ascribed to preeclampsia, maternal hypertension, or IUGR.
		For therapeutic entry: signs and symptoms of preeclampsia or IUGR in the current pregnancy.					
*Byaruhanga 1998 [19]*	230	Previous history of PIH, preeclampsia, especially that occurring at < 32 weeks of gestation, or eclampsia; and pre-existing CHTN.	92%	75 mg	Placebo	20–28 weeks	Preeclampsia; perinatal deaths; and newborn birthweight
*Caritis 1998 [20]*	2539	Pregnant women with pregestational, insulin-treated diabetes mellitus, CHTN, multifetal gestations, or preeclampsia in a previous pregnancy.	98%	60mg	Placebo	16–26 weeks	Preeclampsia, abruptio placentae, preterm birth, infants SGA, neonatal intraventricular hemorrhage, postpartum hemorrhage, and neonatal bleeding.
*Chiaffarino 2004 [21]*	35	CHTN with or without nephropathy, history of severe preeclampsia or eclampsia, history of IUGR, and history of intrauterine fetal death.	87.5%	100 mg	Placebo	<14 weeks	GHTN, preeclampsia, birthweight, mean gestational age at birth.
*Ebrashy 2005 [22]*	139	History of preeclampsia or IUGR, CHTN, positive family history or underlying vascular disorder, maternal age < 20 or > 40 years and gestational diabetes mellitus.	97.8%	75 mg	No treatment	14–16 weeks	Preterm preeclampsia, preeclampsia, IUGR, preterm delivery, Apgar scores, maternal bleeding, and newborn weight.
*ECPPA 1996 [23]*	970	Pregnant women at higher risk of preeclampsia according to a number of reasons including CHTN, primigravity, diabetes mellitus, renal disease, history of preeclampsia or IUGR or evidence of their presence in current pregnancy.	96%	60 mg	Placebo	12–32 weeks	Proteinuric preeclampsia, preterm delivery, IUGR, birthweight, stillbirth and neonatal death; maternal and fetal bleeding.
*Gilani 1994 [24]*	200	History of PIH, BP ≥ 140/90 mmHg on at least two consecutive antenatal visits 15 days apart, history of preeclampsia or eclampsia.	100%	75 mg	Placebo	14 weeks, or after the 1^st^ antenatal visit	Preeclampsia, IUGR, GHTN.
*Golding 1998 [25]*	6049	All nulliparous residents in the parishes of Kingston and St. Andrew in Jamaica.	97%	60 mg	Placebo	12–32 weeks	GHTN, proteinuric preeclampsia, eclampsia, birthweight, preterm delivery, perinatal mortality.
*Haapsamo 2010 [26]*	456	Women age < 40 years, < 4 previous ovarian stimulations, no contraindications for aspirin.	22%	100 mg	Placebo	Pre-pregnancy to delivery if woman becomes pregnant.	PIH, preeclampsia, IUGR, vaginal bleeding during pregnancy, gestational age at delivery, mode of delivery, birthweight, Apgar score, umbilical pH, blood loss in delivery.
*Hauth 1993 [27]*	604	Nulliparous, < 28-years-old.	99%	60 mg	Placebo	20–22 weeks	GHTN, preeclampsia, preterm delivery, PPROM, mode of delivery, fetal death, fetal growth retardation, birth weight, Apgar score.
*Herabutya 1996 [28]*	1348	All normal, nulliparous pregnant women.	90%	60 mg	Placebo	18–24 weeks	Preeclampsia, eclampsia, GHTN.
*Morris 1996 [29]*	102	Nulliparous women with abnormal uteroplacental resistance (S/D > 3.3 or in the presence of an ipsilateral early diastolic notch, an S/D > 3.0 on uterine artery Doppler assessment) at 18 weeks of gestation.	98%	100 mg	Placebo	17–19 weeks	Fetal growth restriction, PIH, preeclampsia, preterm delivery, perinatal death.
*Odibo 2015 [30]*	30	Singleton pregnancy under-going ultrasound examination at 11+0 to13+6 weeks and at high risk for preeclampsia by: CHTN, history of preeclampsia, diabetes, BMI > 30 kg/m^2^, bilateral uterine artery notches, low PAPP-A.	56.6%	81 mg	Placebo	11+0–13+6 weeks	Preeclampsia, GHTN, SGA, preterm birth, stillbirth, antepartum hemorrhage, neonatal death, NICU admission, miscarriage.
*Parazzini 1993 [31]*	920	Prophylactic criteria: age under 18 or over 40 years; mild or moderate CHTN, nephropathy with normal renal function and normal blood pressure, history of PIH with or without proteinuria developing at > 32 weeks of pregnancy, history of IUGR, and current twin pregnancy. Therapeutic criteria: PIH or early signs of IUGR.	94%	50 mg	No Treatment	16–32 weeks	Outcome of pregnancy (induced abortion, spontaneous abortion, delivery), birthweight, duration of gestation, PIH, mode of delivery, NICU admission, GHTN, IUGR.
*Rolnik 2017 [32]*	1,620	An age of 18 years or more, singleton pregnancy, live fetus at the time that scanning was performed and a high risk (>1 in 100) for preterm preeclampsia according to the screening algorithm.	91%	150 mg	Placebo	11–14 weeks	Preterm preeclampsia at < 37 weeks of gestation, adverse outcomes of pregnancy at < 34 weeks, < 37 weeks, and ≥ 37 weeks of gestation (preeclampsia, GHTN, SGA, miscarriage or stillbirth, abruption, spontaneous delivery); death and neonatal complications; neonatal therapy; and poor fetal growth (birthweight < 3^rd^, 5^th^, or 10^th^ percentil
*Rotchell 1998 [33]*	3,641	All pregnant women attending antenatal clinic between 12–32 weeks of gestation.	99%	75 mg	Placebo	12–32 weeks	Proteinuric preeclampsia, other PIH, pregnancy duration, birthweight, stillbirths and neonatal deaths, major neonatal events.
*Subtil 2003 [34]*	3,294	Nulliparous women between 14 and 20 weeks of gestation.	99%	100 mg	Placebo	14–20 weeks	Preeclampsia with proteinuria.
*Talari 2014 [35]*	80	History of preeclampsia, CHTN, positive family history of underlying vascular disorder, gestational diabetes mellitus, or maternal age < 20 years or > 40 years.	70%	80 mg	Placebo	12–16 weeks	Preeclampsia, IUGR, prematurity, mode of delivery, birthweight, and Apgar scores.
*Tewari 1997 [36]*	50	History of CHTN or GHTN, positive rollover test (ROT) at 28–30 weeks of gestation.	70%	50 mg	No treatment	28–30 weeks	GHTN, preterm birth, mean birthweight.
*Vainio 2002 [37]*	86	History of CHTN, familial risk of preeclampsia (mother or sister), gestational diabetes mellitus, maternal age < 20 or > 40 years, history of preeclampsia, IUGR, or previous intrauterine death. Constant bilateral diastolic notch in the uterine arteries at 12 to 14 weeks of gestation on Doppler ultrasound examination.	95.5%	0.5 mg/kg^a^	Placebo	12–14 weeks	PIH, duration of pregnancy, birthweight and IUGR, induction of labor, spontaneous delivery, C-section, postpartum hemorrhage, other fetal outcomes.
*Viinikka 1993 [38]*	197	Women at increased risk for developing preeclampsia: blood pressure before pregnancy > 140/90 mmHg, or severe preeclampsia in prior pregnancy.	95%	50 mg	Placebo	12–18 weeks	Fetal weight, proteinuria > 300 mg/day, exacerbation of pre-existing hypertension (usually > 160/120 mmHg) necessitating the initiation of antihypertensive therapy.
*Villa 2013 [39]*	121	Women with one or more risk factors for preeclampsia, such as: age < 20 or > 40 years, obesity (BMI > 30 kg/m^2^), CHTN, and others.	79.6%	100 mg	Placebo	12+0–13+6 weeks + days	Preeclampsia, GHTN, birthweight standard deviation score, SGA, length of gestation.

GHTN, gestational hypertension; CHTN, chronic hypertension; IUGR, intrauterine growth retardation; PIH, pregnancy-induced hypertension; SGA, small for gestational age; PPROM, preterm premature rapture of membranes; S/D, systolic-diastolic ratio; PAPP-A, pregnancy-associated plasma protein A; BMI, body mass index; NICU, neonatal intensive care unit.

^a^ Daily dose of aspirin was 35–37 mg at the randomization and adjusted at a follow up visit if the weight of the woman exceeded the initial weight by at least 10%.

Results of the bias assessment via the Cochrane Risk of Bias Template are in S1 Fig. None of the included studies had risk of bias for “other bias”; five had risk of bias for selective reporting; eight had risk of bias for incomplete outcome data; eight had risk of bias for blinding of outcome assessment; eight had risk of bias for outcome of blinding participants and personnel; eight had risk of bias for allocation concealment; nine had risk of bias for random sequence generation.

**Fig 2:**
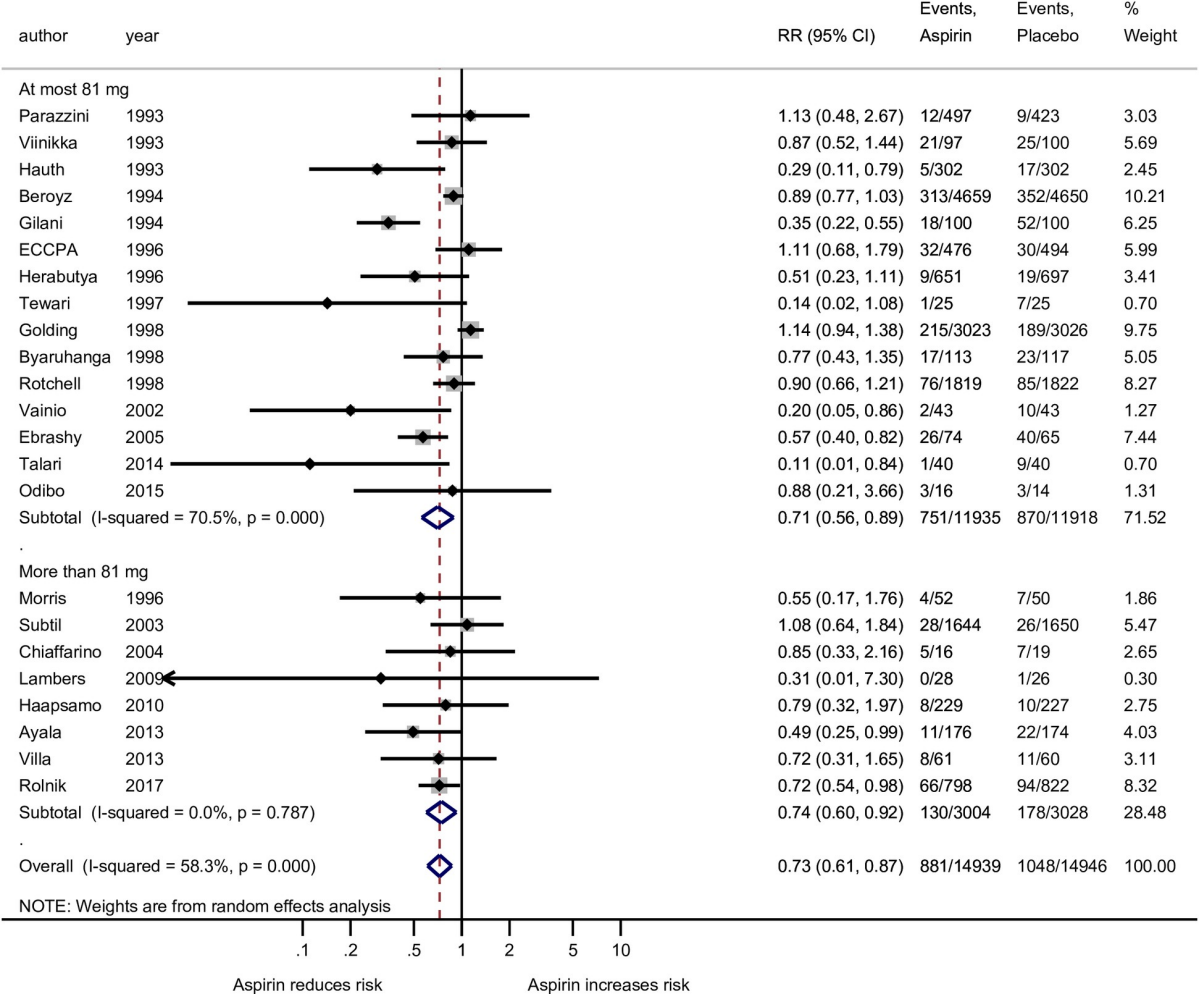
Forest plot of the effect of aspirin on the all gestational age preeclampsia outcome stratified by two groups of aspirin doses: At most 81 mg and more than 81 mg. RR, risk ratio; CI, confidence interval. Black diamond indicates the weight of each study; blue diamond indicates the overall effect of pooled sample; horizontal line indicates 95% confidence interval; solid vertical line indicates no effect.

**Fig 3:**
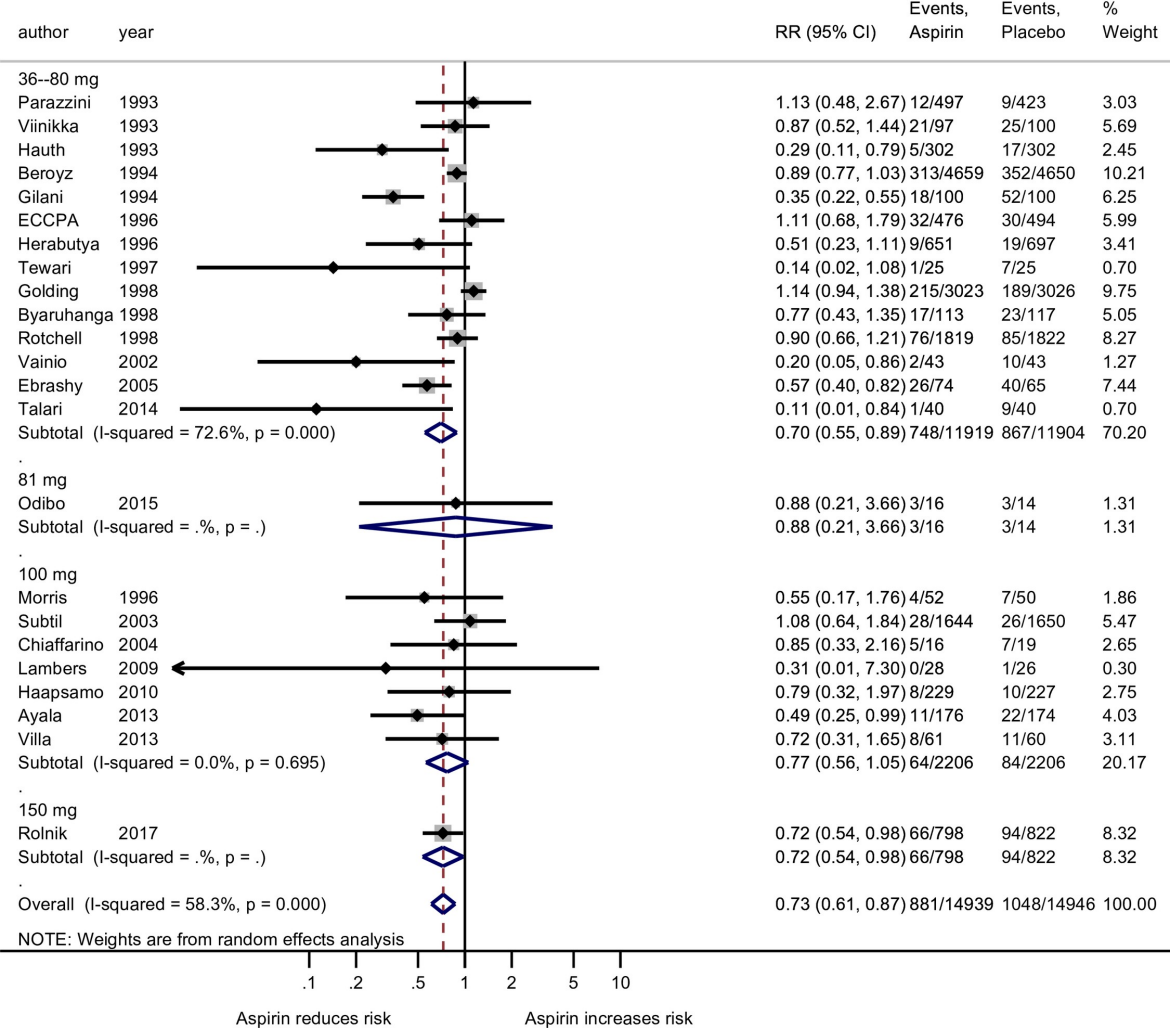
Forest plot of the effect of aspirin on the all gestational age preeclampsia outcome stratified by four groups of aspirin dose: 36–80 mg, 81 mg, 100 mg, and 150 mg. RR, risk ratio; CI, confidence interval. Black diamond indicates the weight of each study; blue diamond indicates the overall effect of pooled sample; horizontal line indicates 95% confidence interval; solid vertical line indicates no effect.

**Fig 4:**
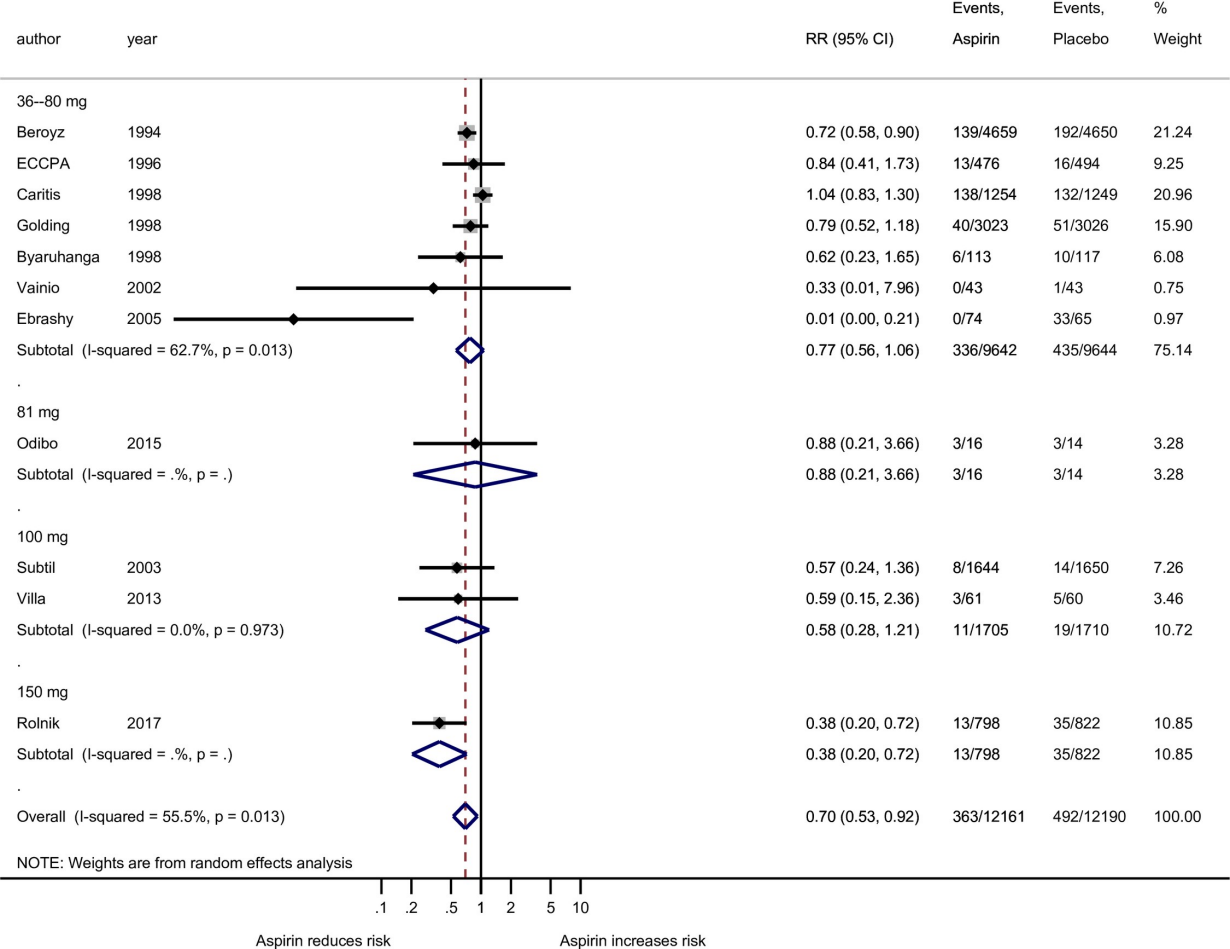
Forest plot of the effect of aspirin on the preterm preeclampsia outcome stratified by four groups of aspirin doses: 36–80 mg, 81 mg, 100 mg, and 150 mg. RR, risk ratio; CI, confidence interval. Black diamond indicates the weight of each study; blue diamond indicates the overall effect of pooled sample; horizontal line indicates 95% confidence interval; solid vertical line indicates no effect.

In our primary analysis involving all gestational age preeclampsia outcome, there was noted heterogeneity among studies using at most 81 mg (*I*^*2*^ = 70.5%; p<0.001) but not in the more than 81 mg group (*I*^*2*^ ≤0.5%; p = 0.725, Fig 2). When the analysis of all gestational age preeclampsia outcome was grouped by the four aspirin dosing groups, there was heterogeneity among the studies using at most 81 mg (*I*^*2*^ = 72.6%; p<0.001) but not in the more than 100 mg group (*I*^*2*^ = 0.0%; p = 0.617, Fig 3). Among the 11 studies used for the preterm preeclampsia analysis there was noted heterogeneity among studies using the at most 81 mg (*I*^*2*^ = 62.7%; p = 0.013) but not in the more than 100 mg group (*I*^*2*^ = <0.0%; p = 0.973, Fig 4). Publication bias cannot be excluded based on the funnel plot (S2A and S2B Fig).

### Effect of aspirin on all gestational age preeclampsia prevention

**Fig 5:**
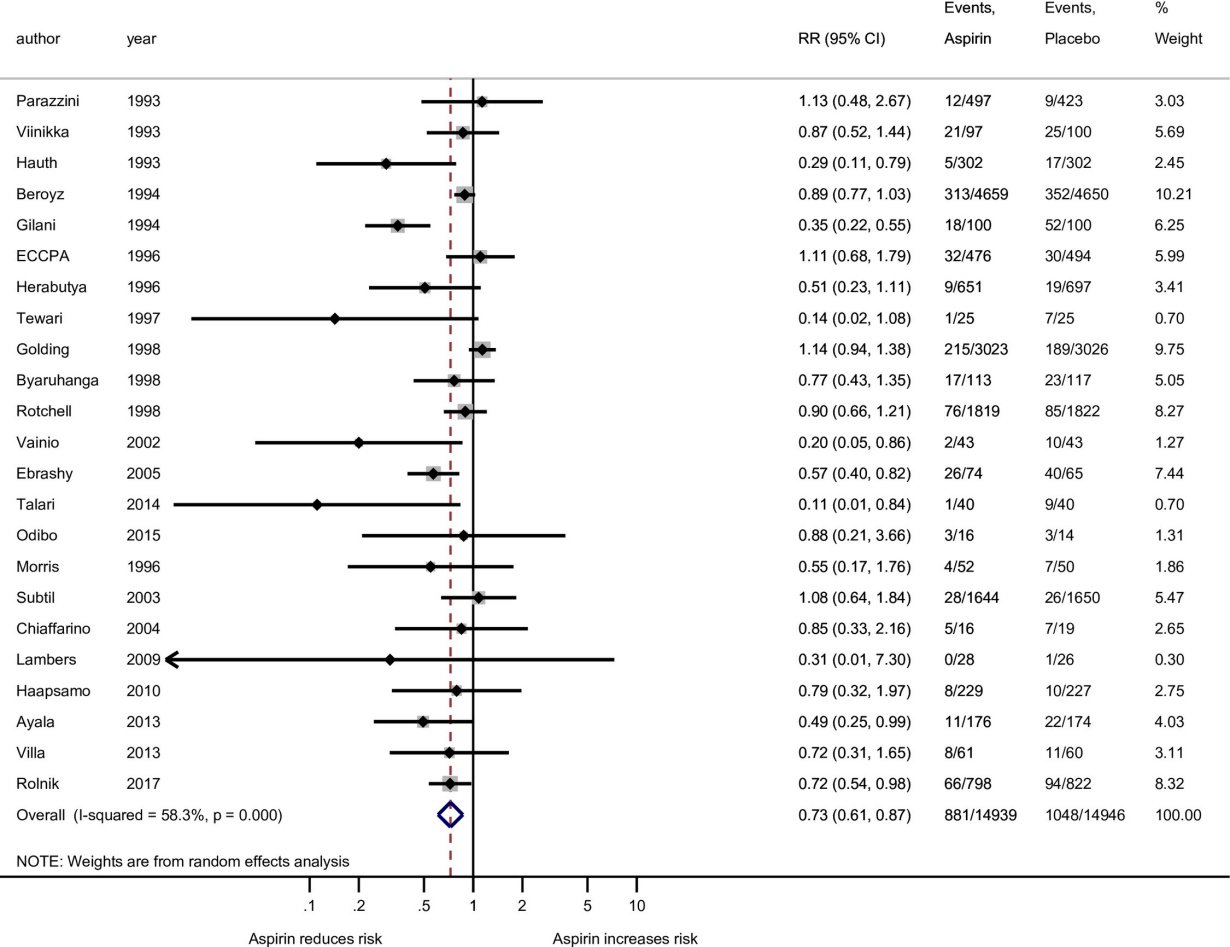
Forest plot of the effect of all aspirin doses on the all gestational age preeclampsia outcome. RR, risk ratio; CI, confidence interval. Black diamond indicates the weight of each study; blue diamond indicates the overall effect of pooled sample; horizontal line indicates 95% confidence interval; solid vertical line indicates no effect.

The meta-analysis of the 22 studies revealed that aspirin regardless of dose brought about a 27% (RR = 0.73; 95% CI: 0.61–0.87; p<0.001) reduction in risk of all gestational age preeclampsia diagnosis compared to placebo (Fig 5).

Of the final 22 studies included, 15 used at most 81 mg of aspirin, one study used exactly 81 mg (the current recommendation according to ACOG and USPSFT guidelines), and 14 other studies used between 36 and 80 mg of aspirin. Six used exactly 100 mg and one study used 150 mg, the highest studied dose for preeclampsia prevention to date. The two groups (at most 81 mg vs higher) each showed significant reductions in risk of preeclampsia due to aspirin. Aspirin at most 81 mg demonstrated a 29% reduction (*RR* = 0.71; 95% CI: 0.56–0.89; p = 0.003) while at more than 81 mg, a 25% reduction (*RR* = 0.75; 95% CI: 0.60–0.93, p = 0.009) in preeclampsia diagnosis (Fig 2). Analyses were also conducted using the risk difference to quantify the effect of aspirin on reduction of preeclampsia (S3 Fig). On this scale, the lower dose of aspirin showed a significant reduction in preeclampsia risk (p = 0.007) while more than 81 mg doses produced a nonsignificant effect (p = 0.115). Those given at most 81 mg saw a 2.6 percentage point (ppt) reduction in risk (95% CI: 0.7–4.4 ppt reduction). This translated to having to treat 38 (95% CI: 23–143) women with lower-dose aspirin to avoid one case of preeclampsia.

The studies accounting for all gestational age preeclampsia outcomes were grouped into the four levels determined by the aspirin doses less than 81 mg, 81 mg, 100 mg, and 150 mg. There was no evidence that exactly 81 mg of aspirin had an effect (RR = 0.88; 95% CI: 0.21–3.66; p = 0.855), and the effect for 100 mg not significant (RR = 0.78; 95% CI: 0.57–1.06; p = 0.113). At the lower and upper ends, there was evidence that aspirin reduces risk and the effect at both extremes was similar, approximately a 28% reduction in risk (RR = 0.70; 95% CI: 0.55–0.89; p = 0.003 and RR = 0.72; 95% CI: 0.54–0.98; p = 0.034; Fig 3). Conclusions were similar when effects are conveyed as simple (additive) reductions in risk: exactly 81 mg and 100 mg each failed to show a robust effect (p = 0.855 and 0.223, respectively), while the lower dose (36–80 mg, p = 0.007; 2.6 (95% CI: 0.7–4.5) ppt reduction) and highest dose (150mg, p = 0.032; 3.2 (95% CI: 0.3–6.1) ppt reduction) each demonstrated protective effects from aspirin (S4 Fig). Those lowest/highest doses correspond to NNT values of 38 (95% CI: 22–143) and 31 (95% CI: 16–333).

### Effect of aspirin on prevention of preterm preeclampsia

**Fig 6:**
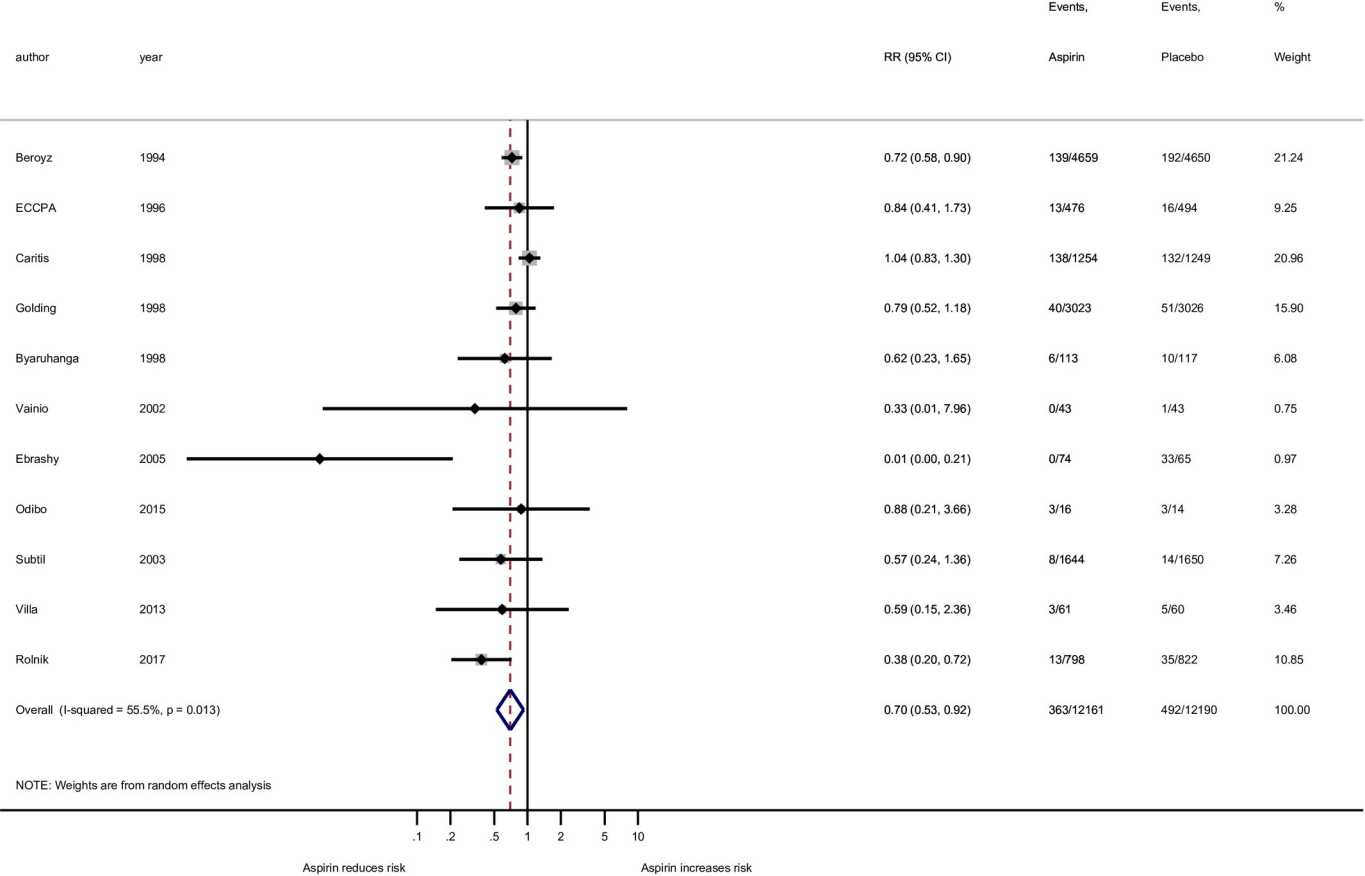
Forest plot of the effect of all aspirin doses on the preterm preeclampsia outcome. RR, risk ratio; CI, confidence interval. Black diamond indicates the weight of each study; blue diamond indicates the overall effect of pooled sample; horizontal line indicates 95% confidence interval; solid vertical line indicates no effect.

Eleven of the 23 studies presented data of preterm preeclampsia outcome. The meta-analysis of the overall effect of aspirin on preterm preeclampsia prevention revealed that aspirin is related to a 30% (RR = 0.70; 95% CI: 0.53–0.92; p = 0.011) reduction in risk of preterm preeclampsia compared to placebo (Fig 6).

**Fig 7:**
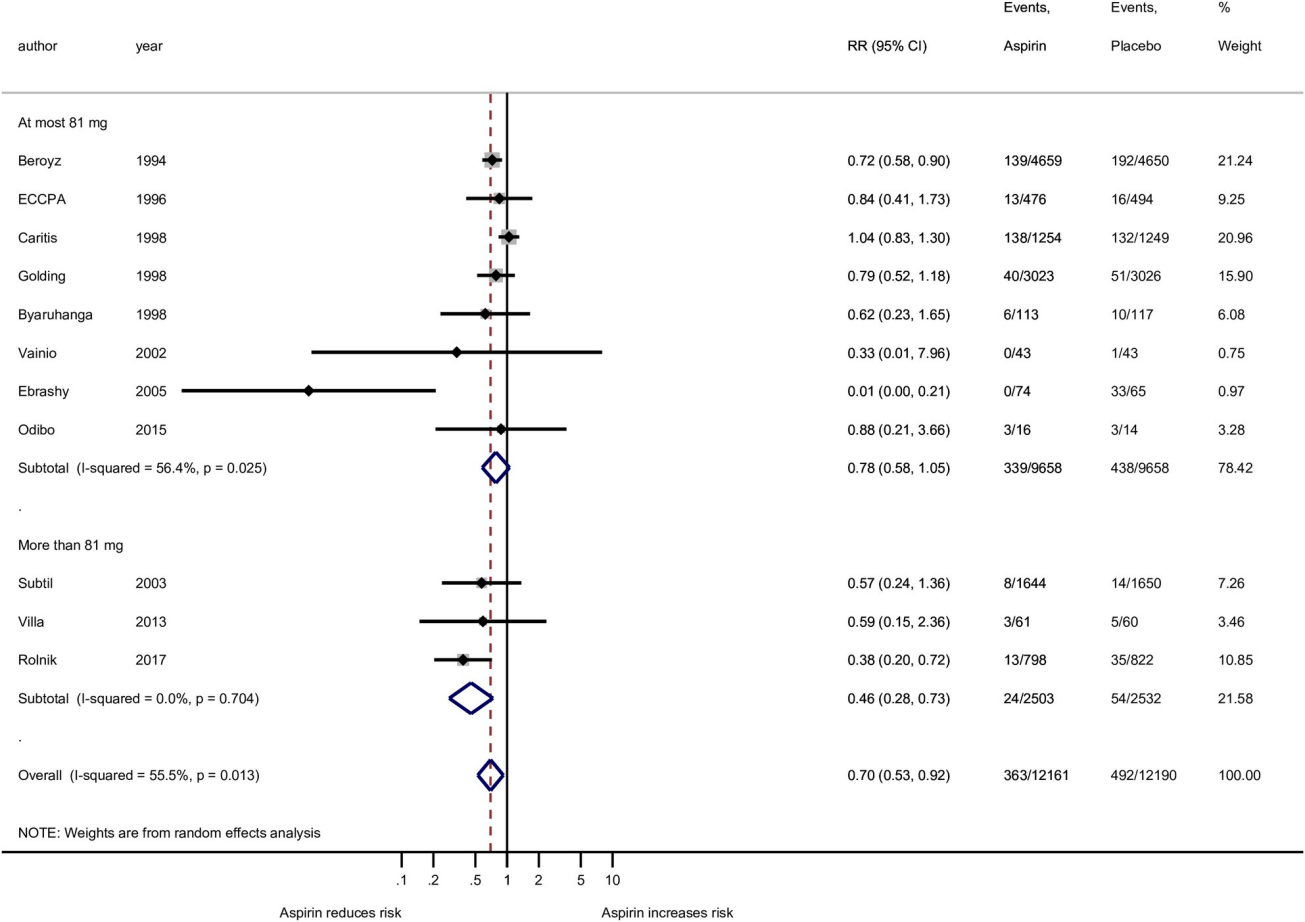
Forest plot of the effect aspirin on the preterm preeclampsia outcome stratified by two groups of aspirin doses: At most 81 mg and more than 81 mg. RR, risk ratio; CI, confidence interval. Black diamond indicates the weight of each study; blue diamond indicates the overall effect of pooled sample; horizontal line indicates 95% confidence interval; solid vertical line indicates no effect.

When grouped according to aspirin dose at most 81 mg (eight studies) and more that 81 mg (three studies), the analysis revealed an insignificant effect of aspirin at most 81 mg (RR = 0.78; 95% CI: 0.58–1.05; p = 0.107), while at more than 81 mg (above currently recommended according to ACOG and USPSFT guidelines) the effect of aspirin was significant and demonstrated 54% (RR = 0.46; 95% CI: 0.28–0.74) reduction in risk of preterm preeclampsia compared to placebo (Fig 7).

Furthermore, studies were grouped according to the four levels of aspirin dose: seven at the low end (36–80 mg), one at exactly 81 mg, two at exactly 100 mg, and one at exactly 150 mg. None of the doses/dose groups that were below 150 mg showed a significant reduction in risk of preterm preeclampsia. The single Rolnik [2017] study demonstrated a convincingly significant risk reduction of 62% (RR = 0.38; 95% CI: 0.20–0.72; p = 0.011) for aspirin compared to placebo (Fig 4). These results should be interpreted with caution considering that the Rolnik study at 150 mg is only a single value providing data for statistical analysis. These same 4 groups of studies, when expressed as risk differences (S5 Fig), showed evidence of protection at the low end (36–80 mg; p = 0.045; 2.4 (95% CI: 0.1–4.6 ppt reduction) and at the highest dose (150 mg; p = 0.002; 2.6 (95% CI: 1.0–4.3 ppt reduction), but not for 81 mg or 100 mg (p = 0.855 and 0.187, respectively). The NNT for 36–80 mg is estimated to be 42 (95% CI: 22–1000) and 39 (95% CI: 23–100) for the 150 mg dose.

## Discussion

All aspirin doses less than 150 mg were ineffective in reducing preterm preeclampsia; however, there was a 62% reduction in risk using 150 mg (RR = 0.38; 95% CI: 0.20–0.72; P = 0.011). The NNT using 150 mg for preterm preeclampsia prevention is 39 (95% CI: 23–100). Dividing the aspirin doses into four groups demonstrated that only less than 81 mg and the 150 mg groups had a significant reduction in risk of all gestational age preeclampsia which was approximately 28% at each end. The NNT using less than 81 mg and 150 mg was 38 (95% CI: 22–143) and 31 (95%CI: 16–333), respectively for preeclampsia prevention at all gestational ages. Again, with the 150 mg being only a single study, it is important to acknowledge that there is a need for more RCTs using 150 mg dosing to increase the statistical power of these conclusions.

Strengths of our review include the number of studies we analyzed compared to other systematic reviews performed on this topic. The last systematic review in February 2018 included 10 studies; we added 13 more randomized controlled trials [6]. We aimed to define a specific dose of aspirin to employ as prevention for preeclampsia versus the current standard of 81 mg, or the generally used and non-specific term: “low-dose aspirin”. Limitations to our review include the potential risk of bias among the articles included, despite our risk assessment and quality measure of each study. We cannot confidently know that all biases were excluded in the methodologic approach to each study that we included in our review. Our percentage point (ppt) risk reduction analysis with the following NNT calculation is limited based on the heterogeneity of the pooled data and should be interpreted cautiously. Our analysis is limited to the congruency among the studies included. Differing outcome measures, cohorts of women, and how each study reported results defined how and what amount of data we could extract. Especially the difference in the gestational age of aspirin therapy initiation in the studies included into this meta-analysis needs to be acknowledged. There is limited data on the optimal aspirin dosing, specifically, of 81 mg compared to 150 mg. This warrants further randomized controlled trials to validate our findings. We recommend future conduction of randomized control trials comparing the efficacy of the daily dosage of 81 mg vs 150 mg aspirin. Analysis of the efficacy of increasing the clinically used dose of aspirin from 81 mg to 150 mg on short and long-term maternal and neonatal outcomes is needed and also recommended.

Since the first evidence of aspirin for reducing the risk of preeclampsia in 1985, many studies have attempted to determine the effect with controversial results. Aspirin dosage remains a topic of debate [12, 13]; however, it is believed there is a dose dependent response.

Currently in the United States, it is recommended in high-risk and considered in moderate-risk women to initiate aspirin prior to 16 weeks and take 81 mg daily until delivery [3, 4, 14]. The USPSTF states that universal aspirin prophylaxis for preeclampsia would save $365 million dollars in direct healthcare costs annually [15]. At best, we interpret all women with moderate or high-risk factors may have approximately a 30% reduction in preeclampsia if they initiate 81 mg of aspirin. When acknowledging the impact of a diagnosis of preterm preeclampsia, this reduction is not sufficient. Our analysis suggests that aspirin doses less than 150 mg may not be reducing the risk of preterm preeclampsia sufficiently. Our results indicate a dose of 150 mg may provide up to 62% reduction of preterm preeclampsia.

The effect of aspirin on COX-dependent prostaglandin synthesis is dose dependent. At lower dosages (less than 100 mg/day) aspirin irreversibly acetylates COX-1, resulting in decreased platelet synthesis of thromboxane-A2 without affecting vascular wall production of prostacyclin, which improves the thromboxane-A2/prostacyclin balance in favor of prostacyclin [12, 13]. At higher doses, aspirin inhibits both COX-1 and COX-2; 1) effectively blocking all prostaglandin production and 2) blocking COX-2 decreases sensitivity to angiotensin II, decreases the activation of the immune system, and decreases oxidative stress. High dose aspirin may be the best option allowing for restoration of the angiogenic balance and improvement in vascular function associated with preeclampsia [16]. Aspirin at low doses has good maternal and fetal safety profiles; however, doses over 100 mg have not been adequately studied. Based on our study, increasing the standard dose of aspirin to 150 mg would provide a substantial reduction in preterm preeclampsia. However, future studies are needed to confirm the mechanism leading to an increased reduction of preterm preeclampsia by use of higher doses of aspirin. In addition, while a higher dosage appears to be the most effective dose for preterm preeclampsia prevention, future studies should validate we are not compromising safety of the mother and baby.

Despite this being based on a single study, our review demonstrated a significant reduction in preterm preeclampsia with 150 mg aspirin compared to all other dosages. We did not find significant differences in risk reduction of preeclampsia at 81 mg in comparison to the other dosages. Randomized trials that compare higher doses of aspirin to 81mg are necessary to complete the totality of evidence.

## Supporting information

S1 Checklist PRISMA 2009 checklist. (DOCX)

S1 Fig Risk of bias assessment according to the Cochrane Collaboration Template. (TIF)

S2 Fig Funnel plot of publication bias. **A:** All gestational age preeclampsia; **B:** Preterm preeclampsia.
(TIF)

S3 Fig Forest plot of the risk difference between aspirin and placebo for all gestational age preeclampsia outcome stratified by two groups of aspirin doses: At most 81 mg and more than 81 mg. (TIF)

S4 Fig Forest plot of the risk difference between aspiring and placebo for all gestational age preeclampsia outcome stratified by four groups of aspirin doses: 36–80 mg, 81 mg, 100 mg, and 150 mg. (TIF)

S5 Fig Forest plot of the risk difference between aspirin and placebo for preterm preeclampsia outcome. (TIF)

## References

[1] Lo JO, Mission J. F., Caughey A. B. Hypertensive Disease of Pregnanct and Maternal Mortality. Curr Opin Obstet Gynecol. 2013;(25):124–32. Epub April 25, 2013. 10.1097/GCO.0b013e32835e0ef5 23403779

[2] Duley L. The Global Impact of Pre-eclampsia and Eclampsia. Seminars in perinatology. 2009;33(3):130–7. 10.1053/j.semperi.2009.02.010 WOS:000266640600002. 19464502

[3] Bulletins ACoP. ACOG Practice Bulletin No. 202 ACOG, The American Journal of Obstetrics and Gynecology2019. Available from: www.acog.org/Womens-Health/Preeclampsia-and-Hypertension-in-Pregnancy?IsMobileSet = false#Providers.

[4] Force USPST. Final Update Summary: Low-Dose Aspirin Use for the Prevention of Morbidity and Mortality From Preeclampsia: Preventive Medication U.S. Preventative Services Task Force2016. Available from: https://www.uspreventiveservicestaskforce.org/Page/Document/UpdateSummaryFinal/low-dose-aspirin-use-for-the-prevention-of-morbidity-and-mortality-from-preeclampsia-preventive-medication.

[5] Rolnik DL, Wright D, Poon LC, O’Gorman N, Syngelaki A, de Paco Matallana C, et al. Aspirin versus Placebo in Pregnancies at High Risk for Preterm Preeclampsia. N Engl J Med. 2017;377(7):613‐22. 10.1056/NEJMoa1704559 CN-01405911. 28657417

[6] Cui YC, Zhu B, Zheng F. Low-dose aspirin at < = 16 weeks of gestation for preventing preeclampsia and its maternal and neonatal adverse outcomes: A systematic review and meta-analysis. Experimental and therapeutic medicine. 2018;15(5):4361–9. 10.3892/etm.2018.5972 WOS:000431850300039. 29725376 PMC5920352

[7] Beaufils M, Uzan S, Donsimoni R, Colau JC. Prevention of pre-eclampsia by early antiplatelet therapy. Lancet. 1985;1(8433):840–2. Epub 1985/04/13. 10.1016/s0140-6736(85)92207-x . 2858710

[8] Peters JL, Sutton AJ, Jones DR, Abrams KR, Rushton L. Contour-enhanced meta-analysis funnel plots help distinguish publication bias from other causes of asymmetry. J Clin Epidemiol. 2008;61(10):991–6. Epub 2008/06/10. 10.1016/j.jclinepi.2007.11.010 . 18538991

[9] Harris R, Bradburn M, Deeks J, Harbord R, Altman D, Sterne J. metan: fixed- and random-effects meta-analysis. Stata Journal. 2008;8(1):3–28.

[10] Harbord RM, Harris RJ, Sterne JAC. Updated tests for small-study effects in meta-analyses. Stata Journal. 2009;9(2):197–210.

[11] Palmer TM, Peters JL, Sutton AJ, Moreno SG. Contour-enhanced funnel plots for meta-analysis. Stata Journal. 2008;8(2):242–54.

[12] Atallah A, Lecarpentier E, Goffinet F, Doret-Dion M, Gaucherand P, Tsatsaris V. Aspirin for Prevention of Preeclampsia. Drugs. 2017;77(17):1819–31. Epub 2017/10/19. 10.1007/s40265-017-0823-0 29039130 PMC5681618

[13] Roberge S, Nicolaides K, Demers S, Hyett J, Chaillet N, Bujold E. The role of aspirin dose on the prevention of preeclampsia and fetal growth restriction: systematic review and meta-analysis. Am J Obstet Gynecol. 2017;216(2):110–20 e6. Epub 2016/09/20. 10.1016/j.ajog.2016.09.076 . 27640943

[14] Cui Y, Zhu B, Zheng F. Low-dose aspirin at <16 weeks of gestation for preventing preeclampsia and its maternal and neonatal adverse outcomes: A systematic review and meta-analysis. Exp Ther Med. 2018;15:4361–9. 10.3892/etm.2018.5972 29725376 PMC5920352

[15] Werner EF, Hauspurg AK, Rouse DJ. A Cost-Benefit Analysis of Low-Dose Aspirin Prophylaxis for the Prevention of Preeclampsia in the United States. Obstetrics and gynecology. 2015;126(6):1242–50. 10.1097/AOG.0000000000001115 WOS:000365302600004. 26551178

[16] Mirabito Colafella KM, Neuman RI, Visser W, Danser AHJ, Versmissen J. Aspirin for the prevention and treatment of pre-eclampsia: A matter of COX-1 and/or COX-2 inhibition? Basic Clin Pharmacol Toxicol. 2019. Epub 2019/08/20. 10.1111/bcpt.13308 . 31420920 PMC7496715

[17] Ayala DE, Ucieda R, Hermida RC. Chronotherapy with low-dose aspirin for prevention of complications in pregnancy. Chronobiol Int. 2013;30(1‐2):260‐79. 10.3109/07420528.2012.717455 CN-00878790. 23004922

[18] Beroyz G, Casale R, Farreiros A, Palermo M, Margulies M, Voto L, et al. Clasp—a Randomized Trial of Low-Dose Aspirin for the Prevention and Treatment of Preeclampsia among 9364 Pregnant-Women. Lancet. 1994;343(8898):619–29. WOS:A1994NA09300006. 7906809

[19] Byaruhanga RN, Chipato T, Rusakaniko S. A randomized controlled trial of low-dose aspirin in women at risk from pre-eclampsia. International Journal of Gynecology & Obstetrics. 1998;60(2):129–35. 10.1016/s0020-7292(97)00257-9 WOS:000072181600003. 9509950

[20] Caspi E, Raziel A, Sherman D, Arieli S, Bukovski I, Weinraub Z. Prevention of Pregnancy-Induced Hypertension in Twins by Early Administration of Low-Dose Aspirin—a Preliminary-Report. Am J Reprod Immunol. 1994;31(1):19–24. 10.1111/j.1600-0897.1994.tb00842.x WOS:A1994MY31400003. 8166943

[21] Chiaffarino F, Parazzini F, Paladini D, Acaia B, Ossola W, Marozio L, et al. A small randomised trial of low-dose aspirin in women at high risk of pre-eclampsia. Eur J Obstet Gynecol Reprod Biol. 2004;112(2):142‐4. CN-00465556. 10.1016/s0301-2115(03)00269-0 14746947

[22] Ebrashy A, Ibrahim M, Marzook A, Yousef D. Usefulness of aspirin therapy in high-risk pregnant women with abnormal uterine artery Doppler ultrasound at 14–16 weeks pregnancy: randomized controlled clinical trial. Croat Med J. 2005;46(5):826‐31. CN-00530130. 16158479

[23] Group EC. ECPPA: Randomised trial of low dose aspirin for the prevention of maternal and fetal complications in high risk pregnant women. 10.1111/j.1471-0528.1996.tb09846.x 8688404

[24] Gilani A, Khan Z. Role of aspirin in management of pregnancy induced hypertension. A study in Pakistani population. Specialist. 1994;10(4):323‐5. CN-00170405.

[25] Golding J, Forrester T, Jones D, McCaw-Binns A, Palmer-Levy M, Wilks R, et al. A randomised trial of low dose aspirin for primiparae in pregnancy. Br J Obstet Gynaecol. 1998;105(3):293–9. 10.1111/j.1471-0528.1998.tb10089.x WOS:000072671500009. 9532989

[26] Haapsamo M, Martikainen H, Tinkanen H, Heinonen S, Nuojua-Huttunen S, Rasanen J. Low-dose aspirin therapy and hypertensive pregnancy complications in unselected IVF and ICSI patients: a randomized, placebo-controlled, double-blind study. Hum Reprod. 2010;25(12):2972–7. 10.1093/humrep/deq286 WOS:000284637500009. 20943705

[27] Hauth J, Goldenberg R, Philips J, Copper R, DuBard M, Cutter G. Low-dose aspirin therapy to prevent preeclampsia: safety considerations. Am J Obstet Gynecol. 1993;168:389. CN-00232172. 10.1016/0002-9378(93)90351-i 8475955

[28] Herabutya Y, Jetsawangsri T, Saropala N. The use of low-dose aspirin to prevent preeclampsia. Int J Gynaecol Obstet. 1996;54(2):177‐8. CN-00141899. 10.1016/0020-7292(96)02701-4 9236320

[29] Morris JM, Fay RA, Ellwood DA, Cook CM, Devonald KJ. A randomized controlled trial of aspirin in patients with abnormal uterine artery blood flow. Obstet Gynecol. 1996;87(1):74–8. Epub 1996/01/01. 10.1016/0029-7844(95)00340-1 . 8532271

[30] Odibo AO, Goetzinger KR, Odibo L, Tuuli MG. Early prediction and aspirin for prevention of pre-eclampsia (EPAPP) study: a randomized controlled trial. Ultrasound Obstet Gynecol. 2015;46(4):414‐8. 10.1002/uog.14889 CN-01255939. 25914193

[31] Parazzini F, Benedetto C, Frusca T, Gregorini G, Bocciolone L, Marozio L, et al. Low-Dose Aspirin in Prevention and Treatment of Intrauterine Growth-Retardation and Pregnancy-Induced Hypertension. Lancet. 1993;341(8842):396–400. WOS:A1993KM41600003. 8094168

[32] Rolnik DL, Wright D, Poon LCY, Syngelaki A, O’Gorman N, de Paco Matallana C, et al. ASPRE trial: performance of screening for preterm pre-eclampsia. Ultrasound Obstet Gynecol. 2017;50(4):492–5. Epub 2017/07/26. 10.1002/uog.18816 . 28741785

[33] Rotchell YE, Cruickshank JK, Gay MP, Griffiths J, Stewart A, Farrell B, et al. Barbados low dose aspirin study in pregnancy (BLASP): a randomised trial for the prevention of pre-eclampsia and its complications. Br J Obstet Gynaecol. 1998;105(3):286–92. 10.1111/j.1471-0528.1998.tb10088.x WOS:000072671500008. 9532988

[34] Subtil D, Goeusse P, Puech F, Lequien P, Biausque S, Breart G, et al. Aspirin, 100 mg daily, did not reduce the risk of pre-eclampsia in nulliparous women. 10.1046/j.1471-0528.2003.02096.x 12742332

[35] Talari H, Mesdaghinia E, Abedzadeh Kalahroudi M. Aspirin and preeclampsia prevention in patients with abnormal uterine artery blood flow. Iranian Red Crescent medical journal. 2014;16(8):e17175. Epub 2014/11/13. 10.5812/ircmj.17175 25389483 PMC4222009

[36] Tewari S, Kaushish R, Sharma S, Gulati N. Role of low dose aspirin in prevention of pregnancy induced hypertension. J Indian Med Assoc. 1997;95(2):43‐4, 7. CN-00144922. 9357241

[37] Vainio M, Kujansuu E, Iso-Mustajarvi M, Maenpaa J. Low dose acetylsalicylic acid in prevention of pregnancy-induced hypertension and intrauterine growth retardation in women with bilateral uterine artery notches. Bjog-an International Journal of Obstetrics and Gynaecology. 2002;109(2):161–7. 10.1111/j.1471-0528.2002.01046.x WOS:000179219900013. 11888098

[38] Viinikka L, Hartikainen-Sorri AL, Lumme R, Hiilesmaa V, Ylikorkala O. Low dose aspirin in hypertensive pregnant women: effect on pregnancy outcome and prostacyclin-thromboxane balance in mother and newborn. Br J Obstet Gynaecol. 1993;100(9):809‐15. CN-00096671. 10.1111/j.1471-0528.1993.tb14304.x 8217999

[39] Villa PM, Kajantie E, Raikkonen K, Pesonen AK, Hamalainen E, Vainio M, et al. Aspirin in the prevention of pre-eclampsia in high-risk women: a randomised placebo-controlled PREDO Trial and a meta-analysis of randomised trials. Bjog-an International Journal of Obstetrics and Gynaecology. 2013;120(1):64–74. 10.1111/j.1471-0528.2012.03493.x WOS:000312304300010. 23126307

