# Supplementary figures and images for: Dose of aspirin to prevent preterm preeclampsia in women with moderate or high-risk factors: A systematic review and meta-analysis
Source: PLoS One. 2021 Mar 9;16(3):e0247782. doi: 10.1371/journal.pone.0247782 (PMC7943022; doi:10.1371/journal.pone.0247782)

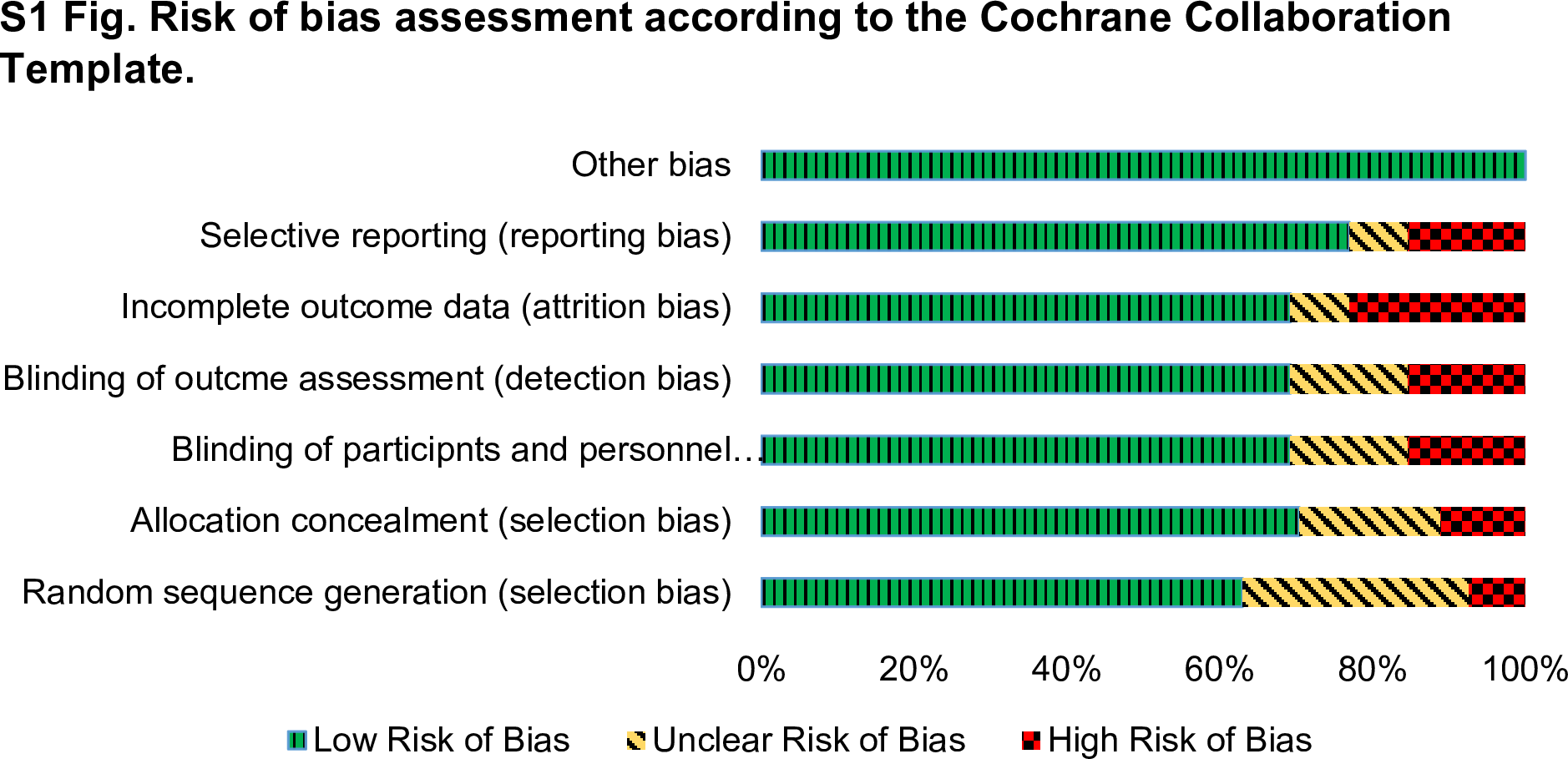

Supplement: S1 Fig — (TIF) [file pone.0247782.s002.tif]

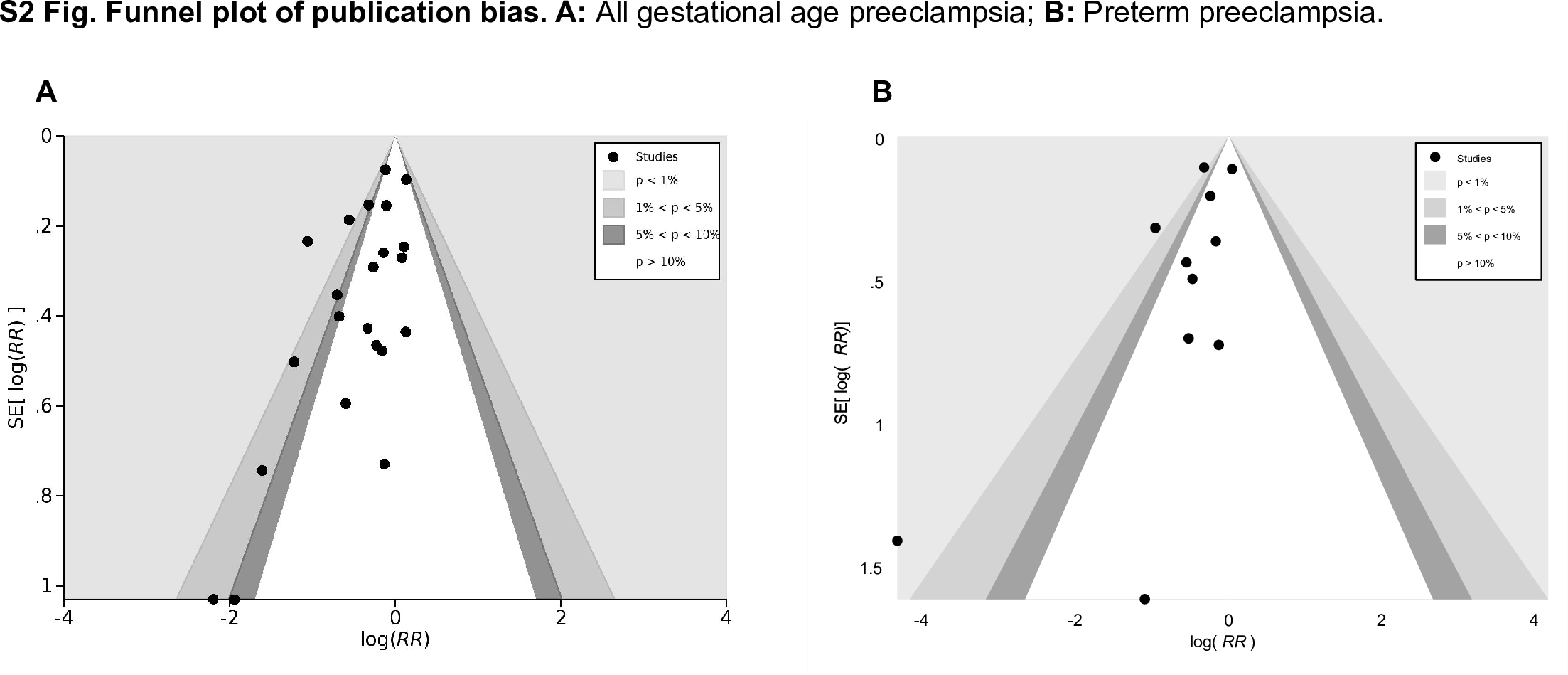

Supplement: S2 Fig — A: All gestational age preeclampsia; B: Preterm preeclampsia. (TIF) [file pone.0247782.s003.tif]

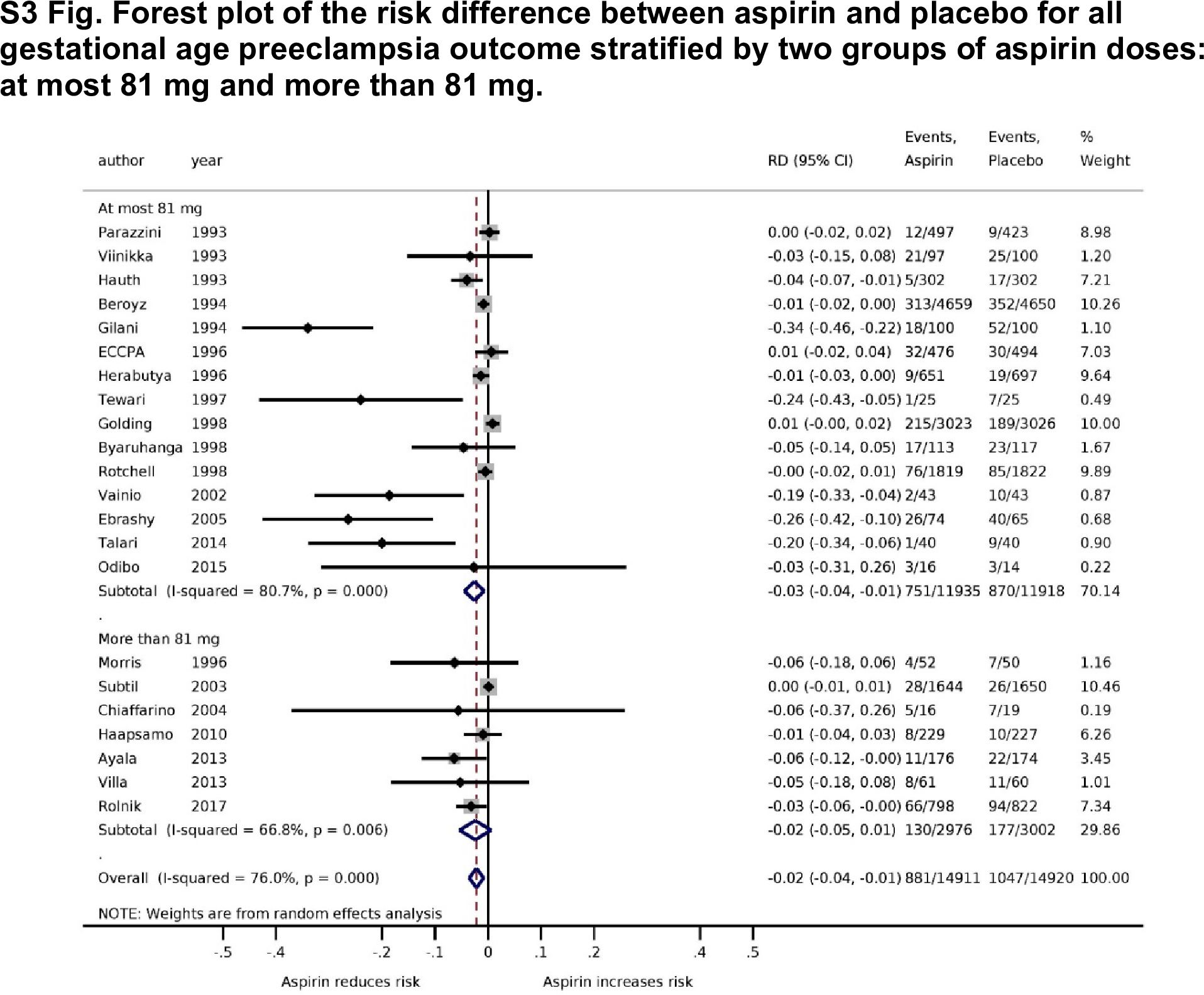

Supplement: S3 Fig — (TIF) [file pone.0247782.s004.tif]

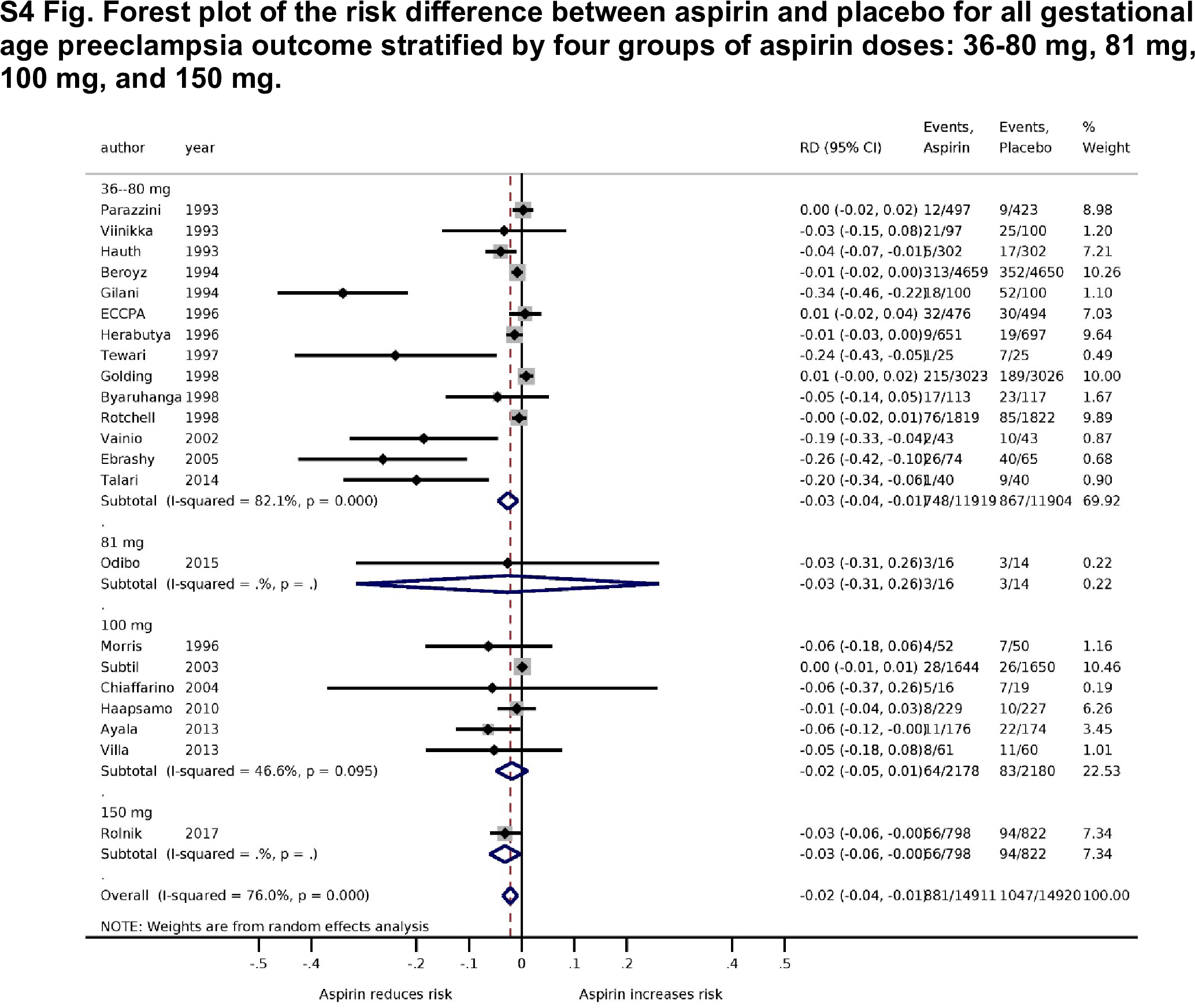

Supplement: S4 Fig — (TIF) [file pone.0247782.s005.tif]

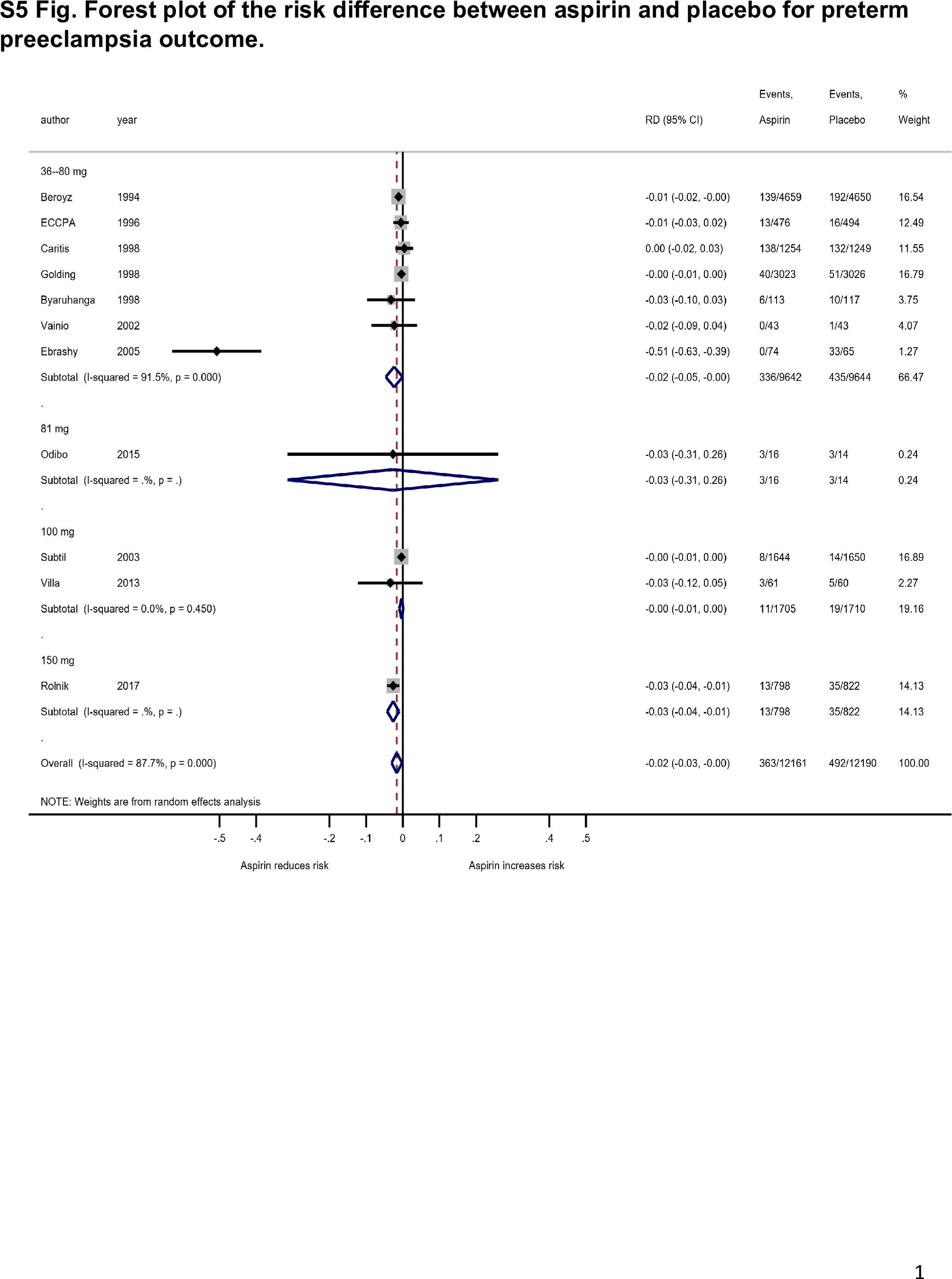

Supplement: S5 Fig — (TIF) [file pone.0247782.s006.tif]
